# C-856-07. Screening for Post-Traumatic Stress Disorder, Depression, and Anxiety in Adult Burn Survivors: A Scoping Review

**DOI:** 10.1093/jbcr/irag033.099

**Published:** 2026-04-06

**Authors:** Amy Boyle, Ervis Musa, Sophie J Huang, Margarita S Elloso, Shahriar Shahrokhi

**Affiliations:** McMaster University, Hamilton, ON; University of Ottawa, Ottawa, ON; Queen's University, Kingston, ON; McMaster University, Hamilton, ON; McMaster University, Hamilton, ON

## Abstract

**Introduction:**

Burn survivors are at an elevated risk of psychological sequelae, including post-traumatic stress disorder (PTSD), depression, and anxiety. Early screening identifies high-risk patients and facilitates timely intervention; however, there is no consensus on the optimal tools for assessment. This scoping review synthesizes existing screening tools for PTSD, depression and anxiety among burn patients.

**Methods:**

This review was conducted according to the Preferred Reporting Items for Systematic Reviews and Meta-Analyses extension for Scoping Reviews. MEDLINE, PubMed and Cochrane were searched from January 2000 to November 2024 for studies measuring symptoms of PTSD, depression, or anxiety in burn survivors over age 18.

**Results:**

Literature search identified 3132 articles, of which 106 studies were included in the final review. In total, 47 unique screening tools were identified: 19 (40%) for PTSD, 18 (38%) for depression, 11 (23%) for anxiety, and 14 (30%) measuring multiple domains. On average, studies used 1.7 tools for 2.2 total measurements. Screening tool results served as an exposure in 23 studies (22%), as an outcome in 82 (77%), and as both in one bidirectional study (1%). Most studies were cross-sectional (n = 52, 49%) or cohort (n = 53, 50%), with one mixed methods design (1%). Sample sizes ranged from 12 to 3953 (mean = 161). Depression was assessed in 72 studies (68%), PTSD in 64 (60%), anxiety in 44 (42%), and multiple domains in 54 (51%). Additional tools not directly related to PTSD, depression, or anxiety were used to explore correlations in 83 studies (78%), for an average of 1.8 additional tools per study.

**Conclusions:**

There is marked variability in the tools and methodologies employed to evaluate psychological outcomes in burn survivors. Numerous studies have investigated the correlations between PTSD, depression, and anxiety symptoms with other clinical or psychosocial factors, suggesting opportunities to strengthen future screening frameworks.

**Applicability of Research to Practice:**

Using validated tools and incorporating strongly correlated variables may improve early detection and treatment of PTSD, depression, and anxiety among burn survivors. This review summarizes available screening tools and the contexts they have been used in, which could inform the development of a standardized approach to screening.

**Funding for the study:**

N/A.

